# ROX index and SpO_2_/FiO_2_ ratio for predicting high-flow nasal cannula failure in hypoxemic COVID-19 patients: A multicenter retrospective study

**DOI:** 10.1371/journal.pone.0268431

**Published:** 2022-05-12

**Authors:** Jin Hyoung Kim, Ae-Rin Baek, Song-I Lee, Won-Young Kim, Yong Sub Na, Bo Young Lee, Gil Myeong Seong, Moon Seong Baek

**Affiliations:** 1 Division of Respiratory and Critical Care Medicine, Department of Internal Medicine, Ulsan University Hospital, University of Ulsan College of Medicine, Ulsan, Republic of Korea; 2 Division of Allergy and Pulmonology, Department of Internal Medicine, Soonchunhyang University Bucheon Hospital, Bucheon, Republic of Korea; 3 Department of Pulmonary and Critical Care Medicine, Chungnam National University Hospital, Daejeon, Republic of Korea; 4 Department of Internal Medicine, Chung-Ang University Hospital, Chung-Ang University College of Medicine, Seoul, Republic of Korea; 5 Department of Pulmonology and Critical Care Medicine, Chosun University Hospital, Gwangju, Republic of Korea; 6 Division of Allergy and Respiratory Diseases Soonchunhyang University Hospital, Seoul, Republic of Korea; 7 Department of Internal Medicine, Jeju National University College of Medicine, Jeju, Republic of Korea; AIIMS: All India Institute of Medical Sciences, INDIA

## Abstract

**Background:**

The ratio of oxygen saturation (ROX) index, defined as the ratio of oxygen saturation (SpO_2_)/fraction of inspired oxygen (FiO_2_) to respiratory rate, can help identify patients with hypoxemic respiratory failure at high risk for intubation following high-flow nasal cannula (HFNC) initiation; however, whether it is effective for predicting intubation in coronavirus disease 2019 (COVID-19) patients receiving HFNC remains unknown. Moreover, the SpO_2_/FiO_2_ ratio has been assessed as a prognostic marker for acute hypoxemic respiratory failure. This study aimed to determine the utility of the ROX index and the SpO_2_/FiO_2_ ratio as predictors of failure in COVID-19 patients who received HFNC.

**Methods:**

This multicenter study was conducted in seven university-affiliated hospitals in Korea. Data of consecutive hospitalized patients diagnosed with COVID-19 between February 10, 2020 and February 28, 2021 were retrospectively reviewed. We calculated the ROX index and the SpO_2_/FiO_2_ ratio at 1 h, 4 h, and 12 h after HFNC initiation. The primary outcome was HFNC failure defined as the need for subsequent intubation despite HFNC application. The receiver operating characteristic curve analysis was used to evaluate discrimination of prediction models for HFNC failure.

**Results:**

Of 1,565 hospitalized COVID-19 patients, 133 who received HFNC were analyzed. Among them, 63 patients (47.4%) were successfully weaned from HFNC, and 70 (52.6%) were intubated. Among patients with HFNC failure, 32 (45.7%) died. The SpO_2_/FiO_2_ ratio at 1 h after HFNC initiation was an important predictor of HFNC failure (AUC 0.762 [0.679–0.846]). The AUCs of SpO_2_/FiO_2_ ratio at 4 h and ROX indices at 1 h and 4 h were 0.733 (0.640–0.826), 0.697 (0.597–0.798), and 0.682 (0.583–0.781), respectively. Multivariable analysis showed that the patients aged ≥70 years are 3.4 times more likely to experience HFNC failure than those aged <70 years (HR 3.367 [1.358–8.349], *p* = 0.009). The SpO_2_/FiO_2_ ratio (HR 0.983 [0.972–0.994], *p* = 0.003) at 1 h was significantly associated with HFNC failure.

**Conclusions:**

The SpO_2_/FiO_2_ ratio following HFNC initiation was an acceptable predictor of HFNC failure. The SpO_2_/FiO_2_ ratio may be a good prognostic marker for predicting intubation in COVID-9 patients receiving HFNC.

## Introduction

High-flow nasal cannula (HFNC) has been widely used in patients with hypoxemic respiratory failure. HFNC could reduce the rate of endotracheal intubation in patients with acute respiratory failure compared with conventional oxygen therapy [1–3]. However, failure of HFNC may cause delayed intubation and increased mortality [4]. Therefore, predicting HFNC failure and determining the appropriate timing of endotracheal intubation are important strategies for HFNC treatment.

The ratio of oxygen saturation (ROX) index, defined as the ratio of oxygen saturation (SpO_2_)/fraction of inspired oxygen (FiO_2_) to respiratory rate, has been proposed to detect HFNC failure [5]. Roca et al. [6] reported that an ROX index cut-off of 4.88 measured 12 h after HFNC initiation was associated with a lower risk of need for intubation, suggesting that the ROX index can help identify patients at high risk for intubation. However, whether the ROX index is effective for predicting intubation and whether the cut-off of the ROX index is appropriate in patients with COVID-19 remain unknown.

The SpO_2_/FiO_2_ (SF) ratio, which correlates with the partial pressure of oxygen PaO_2_/FiO_2_ (PF) ratio [7], can also be utilized as a prognostic marker for acute hypoxemic respiratory failure [8]. Notably, the SF ratio has been reported as a reliable factor for predicting failure of HFNC [9] or non-invasive ventilation [10] in clinical practice when arterial blood gas sampling is not readily available. Moreover, unlike traditional respiratory failure, COVID-19 often presents with silent hypoxia, a condition where the patient has no abnormal respiratory pattern despite severe hypoxia [11]; therefore, respiratory rate may not be crucial in predicting HFNC failure in COVID-19. Hence, we hypothesized that the SF ratio had a comparable prediction ability with the ROX index in patients with severe COVID-19. We aimed to determine the predictive function of the ROX index and the SF ratio in COVID-19 patients after HFNC initiation. Furthermore, we evaluated the factors affecting mortality in these patients.

## Methods

### Study design and patients

This multicenter study was conducted at seven university-affiliated hospitals in Korea with nationally designated isolation units. Patients with confirmed severe acute respiratory syndrome coronavirus 2 (SARS-CoV-2) infection were classified based on severity, and patients with moderate to severe infections were admitted to nationally designated treatment facilities. Consecutive hospitalized patients diagnosed with SARS-CoV-2 infection between February 10, 2020, and February 28, 2021, were retrospectively reviewed. We analyzed the electronic medical records of patients aged ≥18 years who received HFNC. As we enrolled patients who received HFNC in the advanced state of the disease, those who received the therapy following discontinuation of mechanical ventilation were not included in the study group. Patients were excluded if they did not receive HFNC, were intubated after 72 hours from HFNC weaning, or had a do-not-intubate order.

This study was approved by the Institutional Review Board of the Chung-Ang University Hospital (approval number 2103-009-19360) and the local institutional review boards of all other participating centers. Informed consent was waived owing to the retrospective nature of the study.

### Data collection and definitions

The following data were collected from electronic medical records of patients with SARS-CoV-2 infection: age, sex, smoking, symptom at admission, body mass index, Charlson comorbidity index, comorbidities, CURB-65 (Confusion, urea nitrogen, respiratory rate, blood pressure, age 65 ≥ years), SOFA (sequential organ failure assessment) score, APACHE (acute physiology and chronic health evaluation) II score, vital signs at admission, duration of fever, laboratory findings, initial chest x-ray findings (normal or unilateral vs. bilateral or multifocal), treatment during hospitalization (remdesivir, antibiotics, antifungal agents, vasopressor, continuous renal replacement therapy, and corticosteroids), length of hospital stay, PF ratio at HFNC application, HFNC duration, and in-hospital mortality.

The decision to initiate HFNC or mechanical ventilation was made by the treating clinical team at each center. According to the Surviving Sepsis Campaign: guidelines on the management of critically ill adults with COVID-19 [12], HFNC is encouraged to use in acute hypoxemic respiratory failure despite conventional oxygen therapy. The Korean Society of Critical Care Medicine published COVID-19 treatment guidelines indicating mechanical ventilation in the following circumstances [13]:

(1) Persistent or recurrent hypoxia: SpO_2_<92% / PaO_2_<60 mmHg (2) Respiratory rate: 30–35 breaths/min (3) Hemodynamic instability (4) Loss of consciousness/mind alteration (5) Treatment failure of HFNC or conventional oxygen devices (6) Cardiac arrest or respiratory arrest (7) Other: the decision of the physician.

Based on the guidelines, the treating clinical team determined the decision of intubation considering the composite assessment of signs of acute respiratory failure. We calculated the ROX index and SpO_2_/FiO_2_ ratio at 1 h, 4 h, and 12 h after HFNC application. Primary outcome was failure of HFNC, which was defined as the need for subsequent intubation and use of mechanical ventilation despite HFNC application [14].

### Statistical analysis

Categorical variables are expressed as the number (percentage) and were compared using Pearson’s chi-square test or Fisher’s exact test. Continuous variables are expressed as the median (interquartile range), and Mann–Whitney *U* test was used for between-group comparisons. The receiver operating characteristic (ROC) curve analysis was used to evaluate discrimination of prediction models for HFNC failure. The sensitivity and specificity of the scores were determined, and the cutoff point corresponded to the maximum of the Youden’s index. Univariate and multivariable logistic regression analyses were performed using the forward selection method to identify the factors associated with HFNC failure and in-hospital mortality.

Candidate variables for inclusion in the multivariable regression model were variables with *p* < 0.1 in the univariate analyses and clinical parameters. Calibrations of the models were evaluated using the Hosmer–Lemeshow goodness-of-fit test. *P*-values of < 0.05 were considered statistically significant. Statistical analyses were performed using the Statistical Package for the Social Sciences (SPSS) version 26.0 (IBM Corporation, Armonk, NY, USA).

## Results

### Patient characteristics

**Fig 1:**
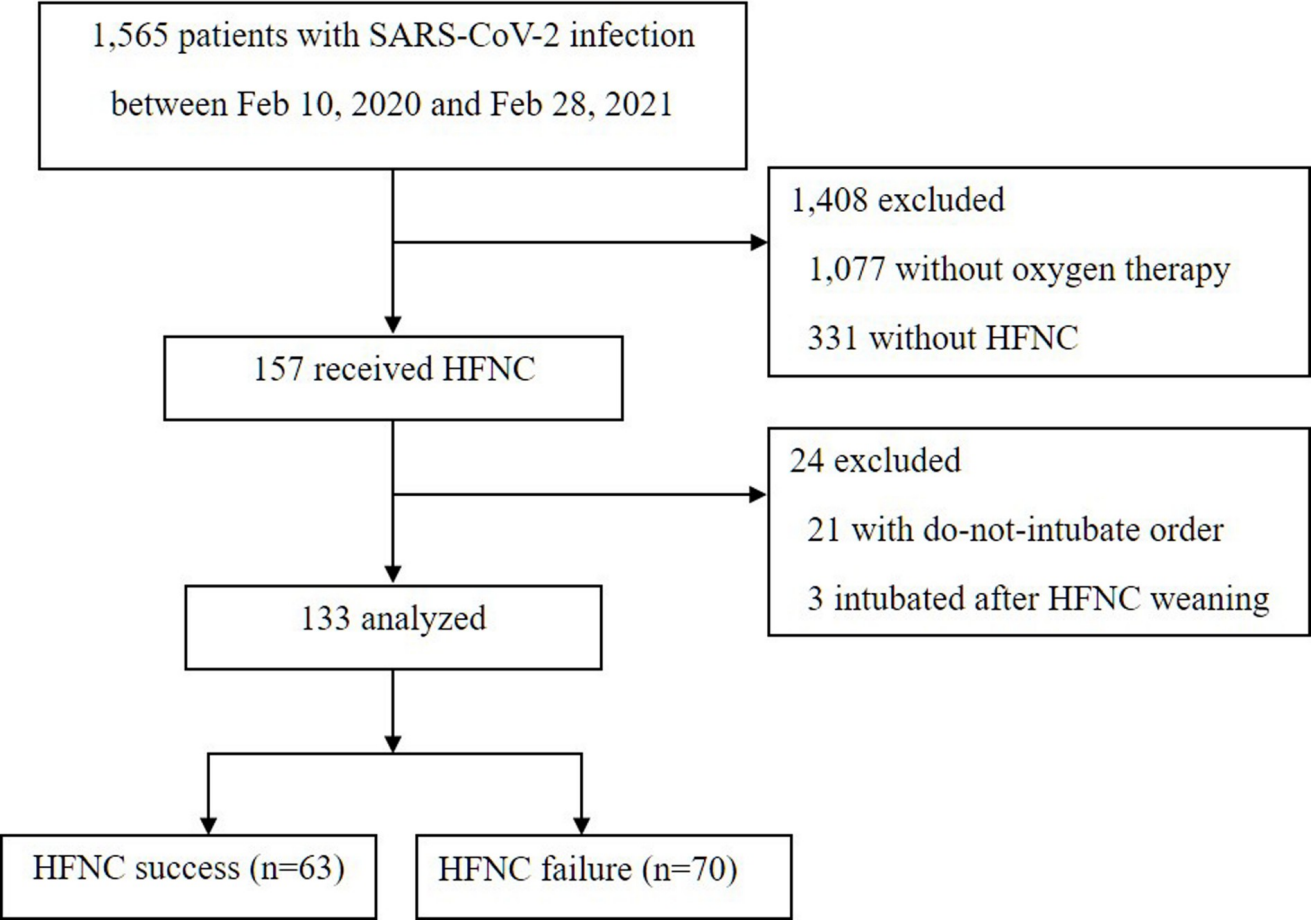
Flowchart of patients with SARS-CoV-2 infection. SARS-CoV-2, severe acute respiratory syndrome coronavirus 2; and HFNC, high-flow nasal cannula.

A total of 1,565 patients with SARS-CoV-2 infection were admitted in seven hospitals, 488 of which received oxygen therapy (Fig 1). HFNC was initiated in 157 patients, and subjects who were intubated after HFNC weaning (*n* = 3) and have a do-not-intubate order (*n* = 21) were excluded from the analysis.

The characteristics of the patients with SARS-CoV-2 infection who were treated with HFNC are presented in Table 1. The HFNC failure group comprised older patients with higher Charlson comorbidity index, SOFA score, and APACHE II score than the HFNC success group. In addition, patients in the HFNC failure group had longer duration of fever, higher incidence of hypertension, higher use of vasopressor, and increased use of continuous renal replacement therapy. No significant differences in initial vital sign measurements, chest x-ray and laboratory results, PF ratio at HFNC application, and the use of corticosteroid were noted between the two groups.

**Table 1:** Characteristics of patients with SARS-CoV-2 infection (N = 133).

Variables	All patients (n = 133)	HFNC success group (n = 63)	HFNC failure group (n = 70)	*P* value
Age, years*	70 (62–78)	68 (58–72)	74 (64–80)	<0.001
Age group, *n* (%)				0.003
<60	23 (15.8)	16 (25.4)	5 (7.1)	
60–69	45 (33.8)	23 (36.5)	22 (31.4)	
70–79	44 (33.1)	19 (30.2)	25 (35.7)	
≥80	23 (17.3)	5 (7.9)	18 (25.7)	
Male, *n* (%)	79 (59.4)	38 (60.3)	41 (58.6)	0.838
Smoking, *n* (%)	25 (18.8)	13 (20.6)	12 (17.1)	0.607
Symptom at admission, *n* (%)	130 (97.7)	61 (96.8)	69 (98.6)	0.603
Body mass index, kg/m^2^*	24.8 (22.4–27.6)	24.8 (22.7–27.6)	25.4 (21.8–27.8)	0.748
Charlson Comorbidity Index*	3 (2–4)	3 (2–4)	4 (2–4.3)	0.001
Comorbidity, *n* (%)				
Hypertension	75 (56.4)	28 (44.4)	47 (67.1)	0.008
Diabetes	42 (31.6)	18 (28.6)	24 (34.3)	0.479
Chronic lung disease	11 (8.3)	5 (7.9)	6 (8.6)	0.894
Chronic kidney disease	5 (3.8)	2 (3.2)	3 (4.3)	1.000
Chronic liver disease	4 (3.0)	1 (1.6)	3 (4.3)	0.621
Cardiovascular disease	10 (7.5)	5 (7.9)	5 (7.1)	1.000
Neurologic disease	4 (3.0)	2 (3.2)	2 (2.9)	1.000
Malignancy	8 (6.0)	2 (3.2)	6 (8.6)	0.280
Scoring systems*				
CURB-65	1 (1–2)	1 (0–2)	2 (1–2)	0.001
SOFA score	3 (1–5)	2 (1–3)	4 (2–7)	<0.001
APACHE II score	9 (7–12)	8 (6–11)	11 (8–13.3)	0.001
Vital signs*				
Systolic blood pressure, mmHg	133 (122–150)	132 (120–148)	135 (123–151)	0.439
Diastolic blood pressure, mmHg	77 (69–86)	77 (70–84)	78 (67–90)	0.806
Heart rate, min^-1^	84 (73–95)	82 (68–90)	89 (75–99)	0.009
Respiratory rate, min^-1^	20 (20–24)	20 (20–22)	20 (20–24)	0.210
Body temperature, °C	36.7 (36.4–37.5)	36.7 (36.2–37.5)	36.7 (36.4–37.4)	0.618
Oxygen saturation, %	96 (92–98)	96 (93–98)	95 (90–97)	0.110
Glasgow Coma Scale	15 (15–15)	15 (15–15)	15 (15–15)	0.328
Duration of fever, days	2 (0–8)	1 (0–3)	6 (1–11)	<0.001
Laboratory findings*				
White blood cells, ×10^9^/L	6.8 (5.0–10.2)	7.0 (5.5–10.4)	6.5 (4.9–10.2)	0.561
Lymphocytes, ×10^9^/L	0.76 (0.48–1.04)	0.78 (0.55–1.04)	0.75 (0.38–1.08)	0.537
Platelet, ×10^9^/L	182 (133–232)	188 (140–238)	179 (126–221)	0.261
Creatinine, mg/dL	0.79 (0.61–0.97)	0.78 (0.60–0.86)	0.80 (0.67–1.06)	0.005
C-reactive protein, mg/dL	10.3 (5.7–15.2)	9.8 (5.0–13.8)	10.3 (6.0–18.2)	0.507
Procalcitonin, ng/mL	0.15 (0.08–0.30)	0.12 (0.08–0.21)	0.16 (0.08–0.61)	0.001
Chest x-ray, *n* (%)				0.461
Normal or unilateral	24 (18.0)	13 (20.6)	11 (15.7)	
Bilateral or multifocal	109 (82.0)	50 (79.4)	59 (84.3)	
Treatment, *n* (%)				
Remdesivir	74 (55.6)	46 (73.0)	28 (40.0)	<0.001
Antibiotics	88 (66.2)	45 (71.4)	43 (61.4)	0.224
Antifungal agents	20 (15.0)	5 (7.9)	15 (21.4)	0.030
Vasopressor	35 (26.3)	2 (3.2)	33 (47.1)	<0.001
Continuous renal replacement therapy	11 (8.3)	1 (1.6)	10 (14.3)	0.008
Corticosteroid	125 (94.0)	58 (92.1)	67 (95.7)	0.476
Length of hospital stay, days*	22 (16–31)	17 (14–22)	28 (21–50)	<0.001
PaO_2_/FiO_2_ at HFNC application*	166 (107–248)	193 (133–252)	151 (93–248)	0.089
HFNC duration, days*	4 (1–7)	7 (5–10)	2 (1–3)	<0.001
In-hospital mortality, *n* (%)	32 (24.1)	0 (0.0)	32 (45.7)	<0.001

*Data are presented as the median (IQR).

SARS-CoV-2, severe acute respiratory syndrome coronavirus 2; HFNC, high-flow nasal cannula; CURB-65, confusion, urea nitrogen, respiratory rate, blood pressure, age ≥ 65 years; SOFA, Sequential Organ Failure Assessment; APACHE, Acute Physiology and Chronic Health Evaluation; PaO_2_, partial pressure of oxygen; and FiO_2_, fraction of inspired oxygen.

### Prediction of HFNC failure

The univariate analysis revealed that age, Charlson comorbidity index, CURB-65, SOFA score, APACHE II score, hypertension, 1 h SF ratio, 4 h SF ratio, 1 h ROX index, and 4 h ROX index were associated with HFNC failure (Table 2). On the other hand, the multivariable analysis revealed that patients aged ≥70 years are 3.4 times more likely to experience HFNC failure than those aged <70 years (HR 3.367 [95% CI: 1.358–8.349], *p* = 0.009). Moreover, the SF ratio at 1 h (HR 0.983 [95% CI: 0.972–0.994], p = 0.003) was significantly associated with HFNC failure.

**Table 2:** Univariate and multivariable analyses of the predictive factors for HFNC failure.

Variables	Univariate analysis		Multivariable analysis	
	OR (95% CI)	*P* value	OR (95% CI)*	*P* value
Age, years		0.008		0.009
<70	Reference		Reference	
≥70	2.588 (1.285–5.212)		3.367 (1.358–8.349)	
Sex		0.838		
Female	Reference			
Male	0.930 (0.465–1.861)			
Smoking	0.796 (0.333–1.901)	0.607		
Body mass index	0.976 (0.896–1.063)	0.578		
Charlson comorbidity index		0.015		
<3	Reference			
≥3	2.464 (1.188–5.113)			
CURB-65		0.014		
<2	Reference			
≥2	2.452 (1.201–5.005)			
SOFA score		<0.001		
<3	Reference			
≥3	4.026 (1.907–8.500)			
APACHE II score		0.029		
<10	Reference			
≥10	2.167 (1.082–4.340)			
Comorbidity	2.974 (1.332–6.640)	0.664		
Hypertension	2.554 (1.263–5.165)	0.009		
Diabetes	1.304 (0.625–2.724)	0.479		
Chest x-ray				
Normal or unilateral	Reference			
Bilateral or multifocal	1.395 (0.574–3.386)	0.462		
Corticosteroid	1.925 (0.441–8.406)	0.384		
PaO_2_/FiO_2_ at HFNC application	0.999 (0.996–1.002)	0.464		
SpO_2_/FiO_2_ 1 h	0.979 (0.970–0.989)	<0.001	0.983 (0.972–0.994)	0.003
SpO_2_/FiO_2_ 4 h	0.983 (0.973–0.992)	0.001		
ROX index at 1 h	0.801 (0.691–0.929)	0.003		
ROX index at 4 h	0.771 (0.660–0.900)	0.001		

*The clinical variables entered into the model were age, Charlson comorbidity index, CURB-65, SOFA score, APACHE II score, hypertension, chest x-ray, corticosteroid, SpO_2_/FiO_2_ ratio at 1 h, SpO_2_/FiO_2_ ratio at 4 h, ROX index at 1 h, and ROX index at 4 h.

HFNC, high-flow nasal cannula; OR, odds ratio; CI, confidence interval; CURB-65, confusion, urea nitrogen, respiratory rate, blood pressure, age ≥ 65 years; SOFA, Sequential Organ Failure Assessment; APACHE, Acute Physiology and Chronic Health Evaluation; PaO_2,_ partial pressure of oxygen; FiO_2_, fraction of inspired oxygen; SpO_2_, percutaneous oxygen saturation; and ROX, pulse oximetry/fraction of inspired oxygen/respiratory rate.

**Fig 2:**
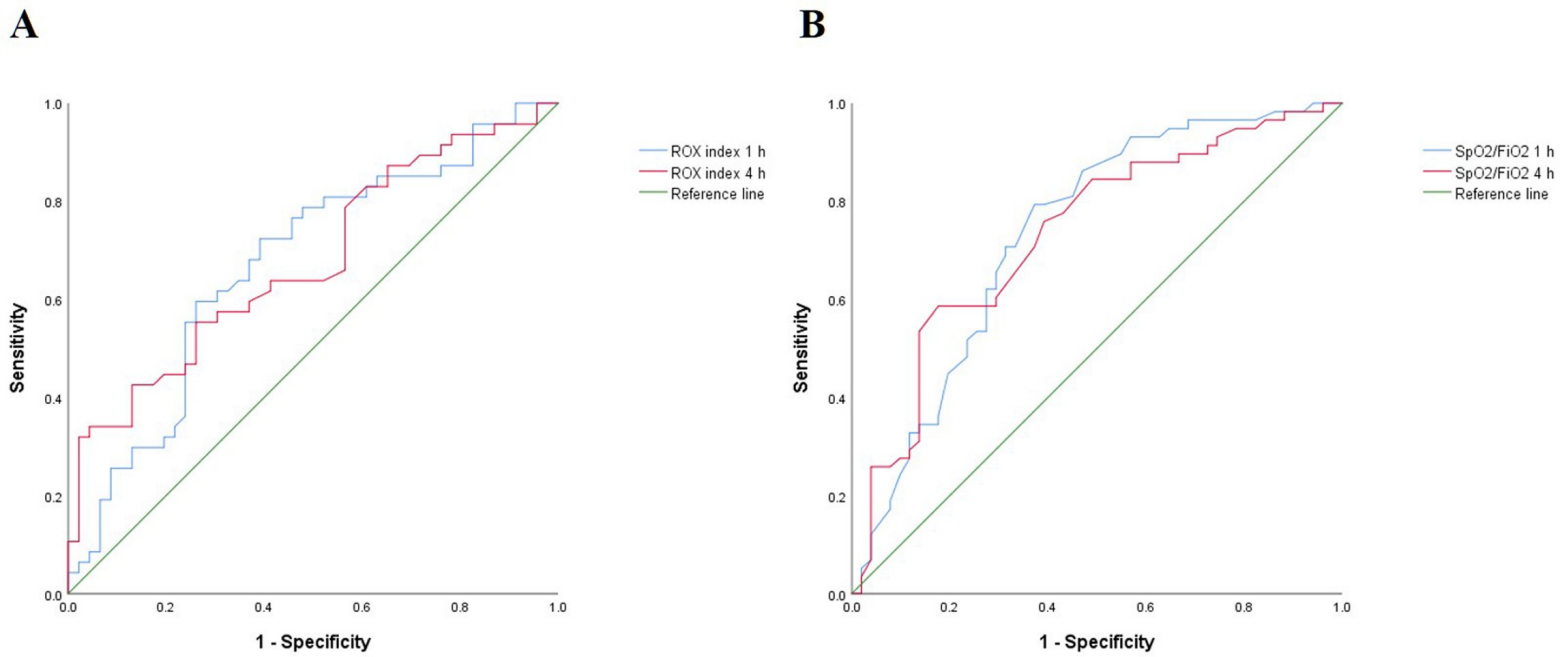
Receiver operator characteristic curves for ROX index and SpO_2_/FiO_2_ ratio as predictor of HFNC failure. HFNC, high-flow nasal cannula; ROX, pulse oximetry/fraction of inspired oxygen/respiratory rate; AUROC, area under the receiver operating characteristic curve; CI = confidence interval; SpO_2_, percutaneous oxygen saturation; and FiO_2_, fraction of inspired oxygen. A. AUROC of ROX indices at 1 h and 4 h were 0.697 (95% CI: 0.597–0.798) and 0.682 (95% CI: 0.583–0.781), respectively. B. AUROC of SpO_2_/FiO_2_ ratios at 1 h and 4 h were 0.762 (95% CI: 0.679–0.846) and 0.733 (95% CI: 0.640–0.826), respectively.

Changes in respiratory variables during HFNC are shown in Table 3. The ROX index and the SF ratio after HFNC initiation were significantly higher in HFNC success group. The SF ratio at 1 h after HFNC initiation was an important predictor of HFNC (AUC 0.762; 95% confidence interval [CI]: 0.679–0.846) (Fig 2). The AUCs of the SF ratio at 4 h and the ROX indices at 1 h and 4 h after HFNC initiation were 0.733 (95% CI: 0.640–0.826), 0.697 (95% CI: 0.597–0.798), and 0.682 (95% CI: 0.583–0.781), respectively.

**Table 3:** Changes in respiratory variables of patients during HFNC (N = 133).

	All patients (N = 133)	HFNC success group (n = 63)	HFNC failure group (n = 70)	*P* value
**ROX index**				
1 hour (n = 107)	7.94 (5.95–10.00)	8.77 (7.66–11.06)	6.85 (5.40–8.43)	<0.001
4 hours (n = 110)	8.29 (6.65–10.08)	9.33 (7.25–12.17)	7.67 (5.80–9.49)	0.001
12 h (n = 90)	9.07 (7.30–11.03)	9.70 (7.56–11.94)	8.64 (6.46–10.06)	0.061
**SpO**_**2**_**/FiO**_**2**_ **ratio**				
1 hour (n = 125)	167 (151–203)	196 (167–236)	160 (120–186)	<0.001
4 hours (n = 115)	167 (155–198)	194 (163–256)	159 (127–188)	<0.001
12 h (n = 96)	190 (158–200)	196 (162–236)	163 (135–193)	0.003
**SpO**_**2**_				
1 hour (n = 125)	96 (93–98)	97 (95–99)	95 (91–97)	0.004
4 hours (n = 116)	97 (95–99)	98 (96–99)	95 (93.5–98)	<0.001
12 h (n = 96)	97 (95–99)	98 (96–99)	96 (95–98)	0.069
**FiO**_**2**_				
1 hour (n = 125)	55 (45–60)	50 (40–60)	60 (50–80)	<0.001
4 hours (n = 116)	60 (50–60)	50 (40–60)	60 (50–80)	<0.001
12 h (n = 102)	50 (40–60)	50 (40–60)	53 (43–68)	0.251
**Respiratory rate**				
1 hour (n = 107)	21 (20–24)	21 (20–25)	21 (20–24)	0.909
4 hours (n = 110)	20 (20–24)	20 (18.5–23)	20 (20–24)	0.305
12 h (n = 90)	20 (18–23)	20 (19–23)	20 (18–23)	0.882

Data are presented as the median (IQR).

HFNC, high-flow nasal cannula; ROX, pulse oximetry/fraction of inspired oxygen/respiratory rate; SpO_2_, percutaneous oxygen saturation; and FiO_2_, fraction of inspired oxygen.

An SF ratio at 1 h after HFNC initiation <166 had a sensitivity of 79.3% and a specificity of 37.3% for predicting HFNC failure (S1 Table). An ROX index at 1 h after HFNC initiation <8.54 had a sensitivity of 59.6% and a specificity of 26.1% for predicting HFNC failure. The AUCs of the ROX index and the SF ratio at 12 h after HFNC initiation were 0.619 (CI: 0.500–0.738) and 0.686 (CI: 0.575–0.797), respectively.

### Predictors of mortality

Age, Charlson comorbidity index, CURB-65, SOFA score, and APACHE II score were factors associated with in-hospital mortality in patients treated with HFNC as revealed by the univariate analysis (Table 4). According to the multivariable analysis results, the odds of death of patients aged ≥70 years were 4.9 times higher than those aged <70 years (HR 4.867 [95% CI: 1.772–13.364], p = 0.002). Patients with SOFA score ≥3 were 4.4 times more likely to die than those with SOFA score <3 (HR 4.437 [95% CI: 1.770–11.120], *p* = 0.001).

**Table 4:** Univariate and multivariable analyses of the predictive factors for in-hospital mortality.

Variables	Univariate analysis		Multivariate analysis	
	Odds ratio (95% CI)	*P* value	Odds ratio (95% CI)*	*P* value
Age, years		<0.001		0.002
<70	Reference		Reference	
≥70	6.341 (2.398–16.770)		4.867 (1.772–13.364)	
Sex		0.216		
Female	Reference			
Male	0.603 (0.271–1.344)			
Smoking	0.750 (0.257–2.192)	0.599		
Body mass index	0.987 (0.893–1.091)	0.799		
Charlson comorbidity index		0.002		
<3	Reference			
≥3	7.462 (2.133–26.099)			
CURB-65		0.006		
<2	Reference			
≥2	3.143 (1.378–7.170)			
SOFA		<0.001		0.001
<3	Reference		Reference	
≥3	5.771 (2.396–13.898)		4.437 (1.770–11.120)	
APACHE II		0.009		
<10	Reference			
≥10	3.090 (1.327–7.200)			
Hypertension	1.394 (0.617–3.152)	0.425		
DM	1.183 (0.509–2.749)	0.696		
CRP	0.974 (0.922–1.030)	0.361		
CXR grade		0.683		
Normal, unilateral	Reference			
Bilateral, multifocal	1.251 (0.426–3.673)			
Remdesivir	0.741 (0.334–1.646)	0.462		
Steroid	0.947 (0.182–4.943)	0.949		
PaO2/FiO2 at HFNC application	0.998 (0.995–1.002)	0.289		

*Clinical variables entered into the model were age, Charlson Comorbidity Index, CURB-65, SOFA score, APACHE II score, hypertension, chest x-ray, and corticosteroid.

OR, odds ratio; CI, confidence interval; CURB-65, confusion, urea nitrogen, respiratory rate, blood pressure, age ≥ 65 years; SOFA, Sequential Organ Failure Assessment; APACHE, Acute Physiology and Chronic Health Evaluation; PaO_2_, partial pressure of oxygen; and FiO_2_, fraction of inspired oxygen.

## Discussion

In this multicenter cohort study in tertiary hospitals in Korea, we evaluated the factors associated with HFNC failure and mortality in COVID-19 patients requiring HFNC. We found an HFNC failure rate as high as 53% and an overall mortality of HFNC-treated patients of 24% at referral. After HFNC application, the ROX index and SF ratio between the HFNC success group and the HFNC failure group were significantly different. Age and SF ratio at 1 h after HFNC initiation were significantly associated with HFNC failure. Furthermore, the SF ratio at 1 h and 4 h after HFNC initiation had an acceptable predictive ability for HFNC failure. Mortality of COVID-19 patients was significantly associated with older age and higher SOFA score.

Since the advent of SARS-CoV-2, there have been reports on the usefulness of the ROX index to predict intubation risk in COVID-19 patients receiving HFNC [14–19]. Studies have demonstrated that ROX indices at 1 h, 2 h, 4 h, 6 h, 8 h, 12 h, and 24 h after HFNC initiation were consistently lower in the HFNC failure group than in the HFNC success group. The AUCs of the ROX indices at 4 h or 6 h after HFNC initiation ranged from 0.70–0.798 [14, 17, 18]. In our study, the predictive function of the ROX index was not remarkable. Compared with those of previous studies, our cohort included relatively older patients with moderate hypoxemia. In addition, ROX indices after HFNC initiation were higher than those reported in previous studies. We attribute the low predictive power of the ROX index to the rapid worsening of respiratory failure in older patients. In cases of acute deterioration in older COVID-19 patients that is not adequately reflected by the ROX index, more accurate or easily applicable alternatives are needed. Furthermore, as the PaO_2_/FiO_2_ ratio can be more accurate than the SpO_2_/FiO_2_ ratio, a modified ROX index using the PaO_2_/FiO_2_ ratio to respiratory rate may be a better prognostic factor that needs validation in the future [20].

Catoire et al. [21] suggested that the AUCs of the SF ratio for PF ratio of 300 and 400 mmHg were high (0.918 and 0.901), which could be used as a hypoxemia screening tool in the emergency department. Using multivariable logistic regression analysis, Patel et al. [22] found that the SF ratio is a significant predictor of intubation risk in COVID-19- related hypoxemic patients. In our study, the SF ratios after HFNC initiation had an acceptable predictive power (AUC: 0.762 and 0.733). Hu et al. [23] reported that both the ROX index at 6 h and the SF and PF ratios at 6 h are accurate predictors of HFNC failure (AUC: SF ratio of 0.786 and PF ratio of 0.749). Arterial blood gas analysis cannot be performed frequently because COVID-19 patients are isolated in a closed room and the number of healthcare personnel is limited. Moreover, respiratory rate may be difficult to objectively monitor because the accuracy of respiratory rate measurements by healthcare professionals is suboptimal [23]. On the other hand, the SF ratio can be calculated by objectively measuring pulse oximetry and FiO_2_; therefore, we suggest that SF ratios can be a useful tool for predicting intubation in COVID-19 patients.

Whether delayed intubation is associated with higher mortality in COVID-19 patients remains controversial. Physicians managing COVID-19 patients may attempt to avoid intubation whenever possible because of the risk of aerosol dispersion, ventilator-associated pneumonia, or complications, such as unplanned extubation. A recent study revealed no association between time-to-intubation and mortality or further lung injury in critically ill COVID-19 patients [24]. In contrast, Hyman et al. [25] reported that the timing of intubation is significantly associated with mortality, with an adjusted hazard ratio for mortality of 1.03 for each day of delay in intubation. Self-inflicted lung injury associated with delayed intubation could also aggravate lung damage [26], resulting in higher mortality. In addition, 73% of hypoxemia cases and 18% of cardiac arrest cases occurred during emergency intubation of COVID-19 patients [27]. These events are more likely to occur when intubation is performed during aggravated hypoxemia due to delayed intubation. The results of this study indicate that older age and higher SOFA score are important factors for mortality in COVID-19 patients receiving HFNC. Therefore, close observation is required to avoid delayed intubation in older patients with higher SOFA score.

This study has some limitations. First, owing to the retrospective nature of this study, the ROX index may have been calculated inaccurately because of errors in measuring respiratory rate. In addition, respiratory rate can often be neglected owing to time constraints and lack of clinical resources [28]. Therefore, given the availability of limited resources due to the COVID-19 pandemic, our results suggest that the SF ratio can be a useful alternative. Second, the timing of intubation was not standardized at each center. Physicians’ experience may have played a crucial role in the decision of intubation. However, the intubation criteria are not significantly different between COVID-19 and conventional respiratory failure and trained board-certified intensivists oversaw decision-making. Third, this study included relatively small number of patients who received HFNC. We performed a post-hoc power analysis with a sample size of 133 and type 1 error of 0.05, achieving a power of 86% for HFNC failure prediction. The post-hoc power analysis ensures sufficient power of the sample size. Nonetheless, prospective randomized controlled studies with a larger number of patients and standardized protocols are needed in the future.

## Conclusions

The SpO_2_/FiO_2_ ratio following HFNC initiation was an acceptable predictor of HFNC failure. The SpO_2_/FiO_2_ ratio may be a good prognostic marker for intubation in COVID-19 patients receiving HFNC. The timing of intubation during HFNC treatment should be carefully tailored to patients, and close monitoring is needed, particularly in older COVID-19 patients.

## Supporting information

S1 Table Prediction accuracy of the ROX index and SpO_2_/FiO_2_ ratio for HFNC failure. (DOCX)

## Abbreviations

HFNC: high-flow nasal cannula
COVID-19: coronavirus disease 2019
SARS-CoV-2: severe acute respiratory syndrome coronavirus 2
ROC: receiver operating characteristic
SpO_2_: pulse oximetric saturation
PaO_2_: partial pressure of oxygen
FiO_2_: fraction of inspired oxygen
SF: SpO_2_/FiO_2_
PF: PaO_2_/FiO_2_
SOFA: sequential organ failure assessment
APACHE II: acute physiology and chronic health evaluation II
