# Supplementary material for: ROX index and SpO2/FiO2 ratio for predicting high-flow nasal cannula failure in hypoxemic COVID-19 patients: A multicenter retrospective study
Source: PLoS One. 2022 May 12;17(5):e0268431. doi: 10.1371/journal.pone.0268431 (PMC9098056; doi:10.1371/journal.pone.0268431)
Supplement: S1 Table — (DOCX) [file pone.0268431.s001.docx]

**S1 Table. Prediction accuracy of the ROX index and SpO_2_/FiO_2_ ratio for HFNC failure.**

| Variable | AUROC (95% CI) | Threshold | Sensitivity | Specificity |
| --- | --- | --- | --- | --- |
| **ROX index** |  |  |  |  |
| 1 h | 0.697 (0.597–0.798) | 8.54 | 59.6% | 26.1% |
| 4 h | 0.682 (0.583–0.781) | 11.52 | 34.0% | 4.3% |
| 12 h | 0.619 (0.500–0.738) | 9.32 | 57.9% | 30.3% |
| **SpO_2_/FiO_2_ ratio** |  |  |  |  |
| 1 h | 0.762 (0.679–0.846) | 166 | 79.3% | 37.3% |
| 4 h | 0.733 (0.640–0.826) | 191 | 58.6% | 17.6% |
| 12 h | 0.686 (0.575–0.797) | 193 | 56.5% | 23.5% |

Data are shown as the median (IQR).

ROX, pulse oximetry/fraction of inspired oxygen/respiratory rate; HFNC, high-flow nasal cannula; SpO_2_, percutaneous oxygen saturation; FiO_2_, fraction of inspired oxygen; and AUROC, area under the receiver operating characteristic curve.
